# Luminance and thresholding limitations of virtual reality headsets for visual field testing

**DOI:** 10.1371/journal.pone.0332795

**Published:** 2025-09-19

**Authors:** Changseok Lee, Liam Redden, Vivian Eng, Brennan Eadie

**Affiliations:** 1 Department of Ophthalmology & Visual Sciences, Dalhousie University, Halifax, Nova Scotia, Canada; 2 Dalhousie Medical School, Halifax, Nova Scotia, Canada; 3 Eadie Technologies Inc., Halifax, Nova Scotia, Canada; Seirei Hamamatsu General Hospital, JAPAN

## Abstract

**Purpose:**

To investigate the luminance capacity and achievable threshold levels of commercially employed virtual reality (VR) devices for visual field testing.

**Methods:**

This two-part study included (1) a literature review of VR headsets used for perimetry with luminance data extracted from technical specifications in publications and manufacturers; and (2) empirical evaluation of three most employed VR headsets in the literature using a custom virtual testing environment.

**Results:**

Three most employed VR devices for visual field testing were Pico Neo, Oculus Quest, and HTC Vive. The maximum reported luminance was 250 cd/m^2^ for the HTC Vive Pro. Information on luminance measurement was not consistently available, reporting only handheld luminance meters.

Empirical measurements show that handheld luminance meters significantly overestimate luminance compared to standard spectroradiometers. Measured luminance varies significantly across aperture size and decreases for peripheral stimuli up to 30 degrees peripherally. Assuming conventional background of 10 cd/m^2^, the best performance with lowest possible thresholding was with HTC Vive at 16dB, corresponding to luminance of 80 cd/m^2^ centrally. Oculus Quest 2 and Pico Neo 3 had minimum threshold of 20dB.

**Conclusion:**

Commercially available VR devices do not meet luminance requirements or threshold sensitivities for visual field testing. Current VR technology is not designed—nor has the capacity—to threshold at mid-to-low dB ranges, which limits accuracy in diagnosing and monitoring defects seen in glaucoma.

Translational Relevance: This study highlights the technical limitations of current commercially available VR devices for visual field testing and significant variables in evaluating luminance performance in these devices.

## Introduction

Visual field testing is central to the diagnosis and monitoring of glaucoma and other ophthalmological and neurological conditions. Glaucoma is the world’s leading cause of irreversible vision loss [1]. In the 1960s, our understanding of glaucoma leapt forward with the development of semi-automated static perimetry [2]. Over the subsequent decade, advancements in computing led to increased automation of visual field testing and the development of standard automated perimetry (SAP) [2]. Since that time, SAP remains the most common test for the diagnosis and monitoring of glaucoma due to its ease of use, wide availability, and standardization of testing [2,3]

Conventional bowl perimeters, such as the Humphrey Visual Field Analyzer (HFA; Carl Zeiss Meditec, Dublin, CA) and the Octopus (Haag-Streit, Koeniz, Switzerland), are the most commonly used visual field testing devices in eye clinics for the diagnosis and monitoring of glaucoma [4]. These perimeters utilize a Tungsten bulb to produce light stimuli with a maximum luminance of approximately 10,000 Asb (3183 cd/m^2^) and neutral density filters to produce dimmer light at decreasing log values [5]. A typical background luminance is 32 Asb (10 cd/m^2^) [5]. Most major studies in glaucoma have utilized these perimeters, and much has been learned about the diagnosis and monitoring of glaucoma using these standardized systems.

The potential application of head-mounted devices to visual field testing preceded the popularization of VR headsets for gaming, though without significant momentum historically [6,7]. With the resurgence of VR recently, an increasing interest developed in the re-purposing of VR headsets designed for gaming for use in visual field testing. There are several potentially attractive features of visual field testing in a head-mounted environment: increased access to visual field testing, testing in non-conventional environments, improved focus, improved access for those with mobility limitations, and lower cost [8]. These advantages are particularly relevant as glaucoma remains under-diagnosed and under-monitored due to lack of appropriate resources and access [9].

To produce varying luminance of light stimuli, VR headsets use displays that produce pixels within the red, green, and blue (RGB) parameters of values in integers between 0 and 255. To produce white stimuli to simulate white on white perimetry, RGB channels are activated concurrently, which allows 255 white luminance levels.

To date, a multitude of VR-based visual field testing devices have been produced, some of which are marketed commercially with limited data. The natural questions arise: How well do VR headsets mimic the standardized specifications of conventional bowl perimeters? How well do VR-based visual field testing systems perform in their output of thresholding luminance sensitivity across the conventionally-tested field of view? In this paper, we address these questions by (1) reviewing the work that has been conducted to date with regards to luminance of VR perimetry systems, (2) experimentally testing the luminance of the most commonly employed VR devices for perimetry, and (3) modelling how these luminance values impact the thresholding capability of VR devices programmed for visual field testing.

## Methods

### Search strategy

A recent systematic review on VR headsets for perimetry was used as a basis for identifying the devices reviewed in this study [8]. Additionally, a Medline search was conducted using the following terms: “virtual reality,” “VR,” “perimetry,” and “visual field testing” to capture any new devices introduced since the systematic review. Conference abstracts were included.

### Data extraction

Information on the technical specifications of devices was obtained directly from publications. Where these specifications were not available in these publications, these specifications were obtained from the source manufacturer of the device or display. Contrast was calculated using the Weber contrast formula ((*L*max -*L*mean)/*L*mean) method used by Sun et al. and expressed as a percentage [10].

### Hardware & software

Three VR head-mounted displays were evaluated: the HTC Vive, Oculus Quest 2, and Pico Neo 3. For accurate readings, luminance levels were measured using a calibrated CAS 140 CT array spectroradiometer (Instrument Systems, Munich, Germany) connected to a TOP 150 telescopic optical probe (Instrument Systems, Munich, Germany) with a Nikon DX AF-S Nikkor 55–200 mm lens attachment. A preset aperture was mounted on the front of the lens to limit the amount of light that enters the spectrometer. The spectroradiometer was placed at eye position within each VR headset and aligned to the optical centre of the eyepiece. The eye relief distances were 17 mm for HTC Vive, 16 mm for Oculus Quest 2, and 17 mm for Pico Neo 3.

On a Windows 10 PC with 16GB of RAM and i7-6700 processor running SpecWin Pro (v 3.7.2.3100, Instrument Systems, Munich, Germany) and LabVIEW (National Instruments, Austin, TX), we used XILab software (v 1.18.3, Standa Ltd, Lithuania) to control 2 rotary motors on 8SMC5 motor controller that allows for precise movements of the spectrometer sensor. For handheld luminance recordings, a SM208 screen luminance meter, similar to that used by all other studies performed to date, was employed.

A custom virtual testing environment was developed using Unity 2022.3.32f1 (Unity Technologies, San Francisco, CA). Each VR head-mounted device runs its own custom application that connects to a server and presents an evenly white background. Luminance levels were set at maximum using each device’s native settings where available, then adjusted using an interface from the server application running from a Windows 10 PC with 16GB of RAM and a i7-7700 processor.

### Measurements

Unless otherwise stated, RGB values were measured for VR devices at steps of 1, 50, 100, 150, 200, and 255 (0–255 available). For white-on-white perimetry, all RGB channels are on equally and simultaneously. Three trials were performed for each test condition, and the average value was used for analysis. Each trial was reset prior to measurement to account for potential variability in device setup. Luminance measurements were obtained using the spectrometer in a dim room. In select cases, all RGB values were measured. Gamma correction was employed in Unity for luminance measurements.

### Aperture size

The human pupil was modelled using custom 3D-printed apertures of 2, 4, and 8 mm, which were fitted onto the camera lens attachment.

### Non-central measurements

Each VR headset was visually aligned to the center and moved using the stepper motor controller to ensure precision across the field of view at 0, 10, 20, and 30 degrees from center by rotation.

### Handheld luminance meter

SM208 handheld luminance meter was aligned manually to the center of each VR device. RGB levels of 0, 50, 100, 150, 200, and 255 were recorded for all devices.

### Achievable thresholds

Achievable threshold values were calculated under various assumed background luminance levels: 10, 5, and 1 cd/m^2^. An increasing log scale of stimulus values was generated in accordance with the Weber contrast formula: (Lmax−Lmean)/(Lmean). The measured luminance values were cross-referenced with these theoretical values. A dB level was determined to be “achievable” if any RGB level could produce a luminance level within +/-10% of the calculated luminance value required.

### Statistical analyses

All analysis was performed in the program R (Posit, Boston, MA). 95% Confidence Intervals (CIs) and p-values were computed using a Wald *t*-distribution approximation.

## Results

### Review of VR headsets for perimetry

**Fig 1:**
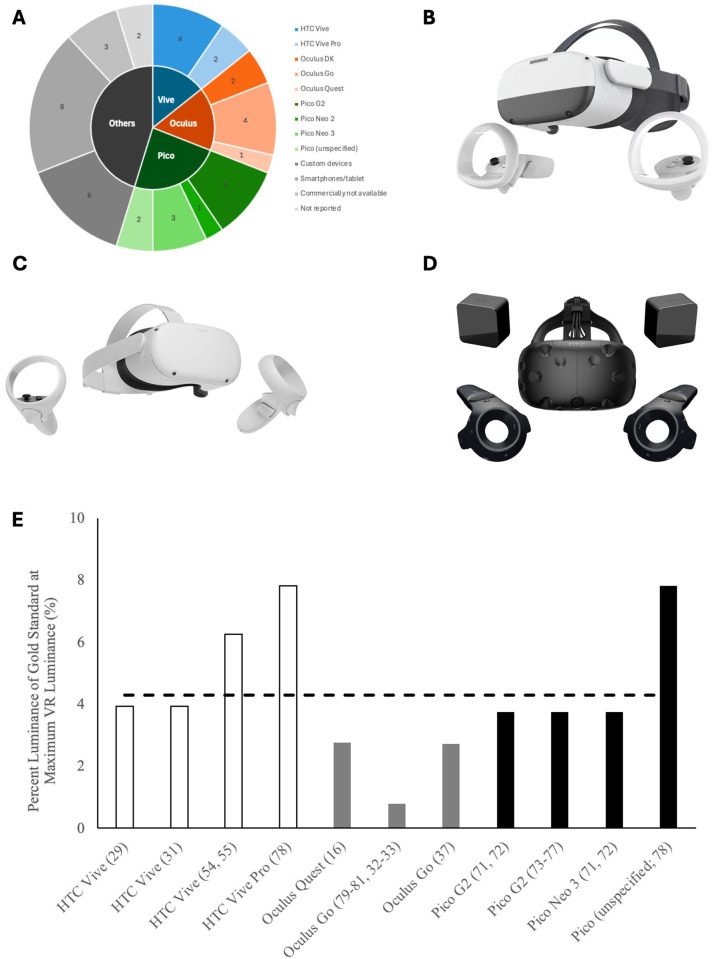
Review of VR devices used for visual field testing in the literature. (A) Type of VR devices used for visual field testing in the literature. Three most commonly used headsets: (B) Pico Neo 3, (C) Oculus Quest 2, and (D) HTC Vive. (E) Percent maximum luminance of VR devices compared to that of conventional perimeters.

Thirty-six devices were reviewed based on a recent systematic review of VR Headsets for perimetry [8]. Two new devices since publication of the systematic review were identified on further search; the Order of Magnitude (OM) and the Radius, for a total of thirty-eight studies and forty-two devices [11–13]. (Fig 1A).

Seven of the forty-two devices (16.67%) utilized a smartphone as the light source. One device (2.38%) utilized a tablet. The remaining 34 devices (80.95%) utilized a built-in screen within the headset. Six devices were custom built devices by the study team. The three most used VR devices were the Pico (10; 23.81%), Oculus (7; 16.67%), and HTC Vive (6; 14.29%) (Fig 1A–D).

Of the forty-two devices, twelve (28.57%) had no luminance data available. For the remaining thirty devices, only commercially available devices were included due to reliability and accuracy of the reported luminance data. We identified seven commercially available devices across eleven studies.

Of the seven commercially available devices, the range of *maximum* luminance (L_max_) was very low: 25–250 cd/m^2^. Percent of HFA L_max_ was calculated relative to conventionally required luminance (3183 cd/m^2^) (Table 1). The mean (SD) L_max_ was 137.41 (±66.11) cd/m^2^ (mean percent L_max_ 4.29%). The device with the lowest L_max_ was the Oculus Go at 25 cd/m^2^ (percent L_max_ 0.78%). The device with the highest L_max_ was the HTC Vive Pro and Pico (unspecified) at 250 cd/m^2^ (percent L_max_ 7.81%). Of note, there was inter-study variability of reported luminance among the same devices (Fig 1E).

**Table 1:** Commercially available devices used for visual field testing reported in the literature, their marketed device name, and reported maximum luminance.

Device Platform	Device Name	Maximum Luminance	Percent of HFA Maximum Luminance	Reference
Pico G2	Virtual Reality Visual Field Device by Virtual Vision	120	3.75	[14–16]
	VisuALL Field Analyzer by Olleyes Inc.	120	3.75	[17–23]
	VirtualField.io	Not reported	Not reported	[24–26]
	Superior-64 Visual Field Test Strategy	Not reported	Not reported	[27]
Pico Neo 2	Order of Magnitude	Not reported	Not reported	[11,12]
Pico Neo 3	Virtual Reality Visual Field Device by Virtual Vision	120	3.75	[14–16]
	VirtualField.io	Not reported	Not reported	[24–26]
	Superior-64 Visual Field Test Strategy	Not reported	Not reported	[27]
Pico (unspecified)	Vive Pro and Pico Headset	250	7.8125	[28]
	Palm Scan VF2000 Virtual Reality Visual Field Analyzer	Not reported	Not reported	[29–31]
Oculus DK	Functions’ Explorer Based on a Virtual Reality Headset (FEX-VRH)	Not reported	Not reported	[32,33]
	SimField Using the Oculus Rift Development Kit 2	Not reported	Not reported	[33,34]
Oculus Go	Vivid Vision Perimetry	25	0.78125	[35–39]
	Virtual Field	87	2.71875	[40,41]
	Order of Magnitude	Not reported	Not reported	[11,12]
	Virtual Reality-Based Oculokinetic Perimetry (VR-OKP)	Not reported	Not reported	[42]
Oculus Quest	Oculus Quest Headset	88	2.75	[43]
HTC Vive	HTC Vive Headset-Embedded Tobii	200	6.25	[44,45]
	Luxie with HTC Vive Pro Eye Headset	125.73	3.9290625	[46]
	Median Cut Hemifield Test (MCHT for the HTC Vive Pro Eye Headset)	125.73	3.9290625	[47]
	Digital Spectacles (DSpecs)	Not reported	Not reported	[48,49]
HTC Vive Pro	Vive Pro and Pico Headset	250	7.8125	[28]
	Virtual Reality Gaze-Contingent Flicker Pupil Perimetry (VRgcFPP) administered on HTC Vive Pro Eye VR headset	Not reported	Not reported	[50,51]

Information on the method of luminance measurement was not consistently available. Among the VR devices with standardized luminance measurements, three handheld luminance meters were named: Mavomonitor USB Luminance Meter, LX101A Light Meter Luxmeter (HTC, Taoyuan City, Taiwan), and Extech EA31 EasyView Light Meter. Luminance for the other VR devices were measured using un-named photometers, not reported, or extrapolated from smartphone luminance used within the headset.

### Empirical evaluation of VR devices

**Fig 2:**
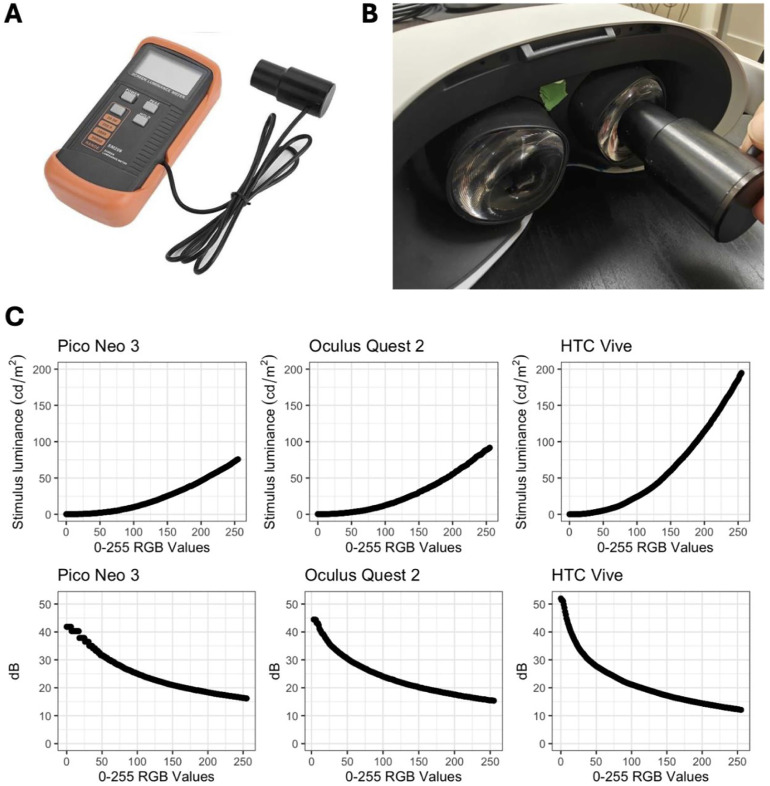
Handheld luminance meter setup and measurment of VR headsets. (A) SM208 handheld luminance meter. (B) Handheld luminance meter on an Oculus Quest 2 headset. (C) Handheld luminance meter measurements on Pico Neo 3, Oculus Quest 2, and HTC Vive at RGB values of 0 to 255 (top row), with corresponding dB values assuming background luminance of 10 cd/m^2^ (bottom row).

**Fig 3:**
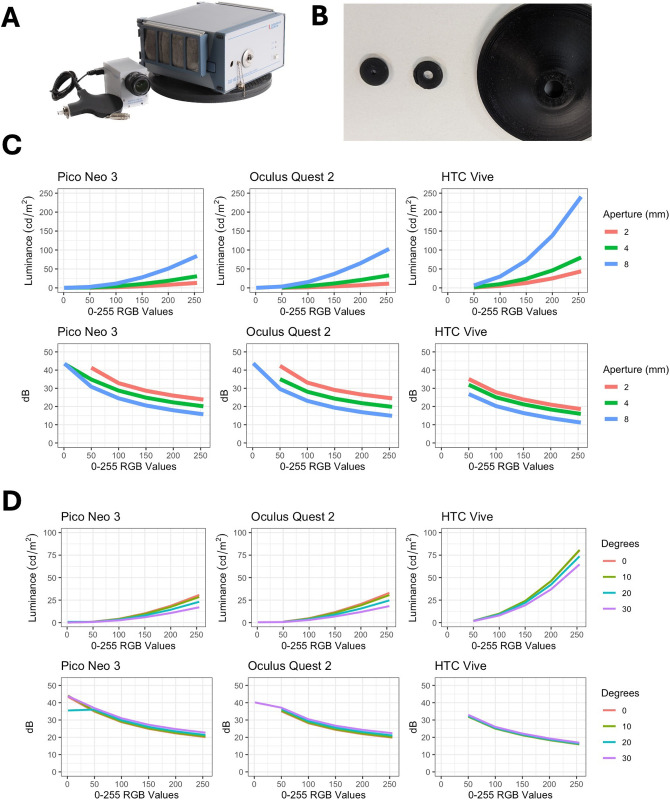
Spectroradiometer setup and luminance measurements of VR headsets across RGB values and eccentricities. (A) CAS140 spectroradiometer with TOP 150 optical probe. (B) Custom 3D-printed 2 mm, 4 mm, and 8 mm apertures. (C) Spectroradiometer measurements of Pico Neo 3, Oculus Quest 2, and HTC Vive at RGB values of 0 to 255 using 2 mm, 4 mm and 8 mm apertures (top row), with corresponding dB values assuming background luminance of 10 cd/m^2^ (bottom row). (D) Spectroradiometer measurements of Pico Neo 3, Oculus Quest 2, and HTC Vive at RGB values of 0 to 255 at 0, 10, 20 and 30 degrees (top row), with corresponding dB values assuming background luminance of 10 cd/m^2^ (bottom row).

To empirically assess luminance performance, three commonly used VR devices were acquired and tested with a handheld luminance meter (Fig 2A, B) and high-resolution spectroradiometer (Fig 3A): Pico Neo 3, Oculus Quest 2, and HTC Vive. Custom apertures of 2, 4, and 8 mm were 3D-printed for experimental luminance measurements (Fig 3B). Linear mixed models showed that aperture size had a significant positive effect on luminance (β = 7.11, *p* < .001, marginal *R*² = 0.69), while the effect of RGB value squared was non-significant (β = 0.00121, *p* = 0.134) (Fig 3C).

Using a 4 mm aperture size, luminance recordings were taken at 0, 10, 20, and 30 degrees from center (Fig 3D) with the spectroradiometer at 0−255 RGB values. Luminance decreased with increasing degrees from the center (β = −0.17, *p* < .001, marginal *R*² = 0.96), with a significant positive effect of RGB value squared (β = 0.000487, *p* = 0.004). The highest L_max_ level measured was HTC Vive at 0 degrees with 80 cd/m^2^, and the lowest L_max_ was Pico Neo 3 at 30 degrees with 25 cd/m^2^.

Next, with 4 mm aperture size, the handheld luminance meter was used for measurements at RGB levels of 1, 50, 100, 150, 200, and 255 for all three VR devices at 0 degrees (Fig 2C). An RGB value of 1 returned variably low luminance recordings values to the 4^th^ decimal place that were at times negative and therefore could not consistently be reported for calculations involving contrast. The measurements are represented in luminance and decibels (dB). The handheld meter measures significantly higher luminance values (and lower dB) compared to the spectroradiometer across all tested RGB levels in the three VR devices (Fig 3D; **0 degrees**) (β = −24.35, *p* < .001, marginal *R*² = 1.00), while the effects of RGB value squared (β = −0.35, *p* = 0.928), and the interaction between tool and degrees (β = −0.17, *p* < .001) were also included in this model.

**Fig 4:**
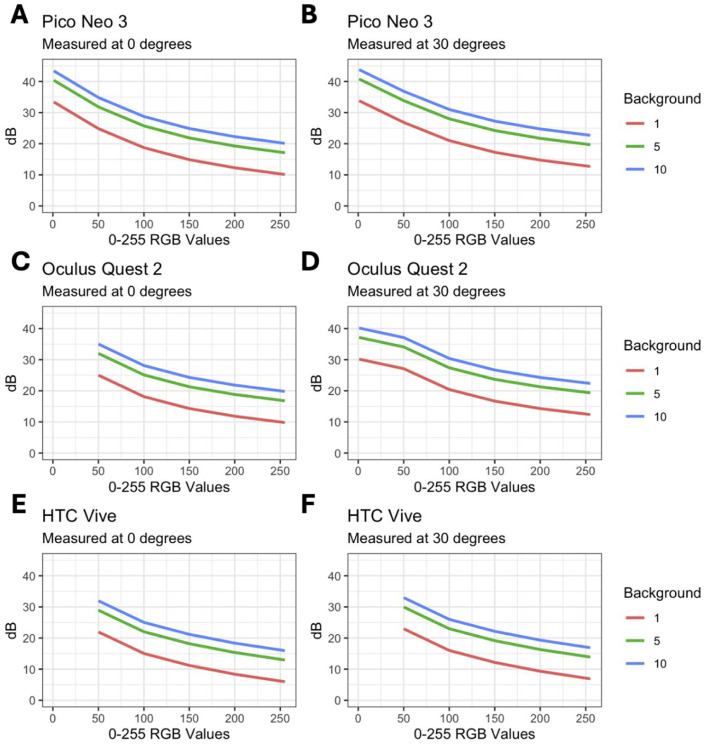
Calculated dB values for VR headsets with varying background luminance and eccentricities. (A) Calculated dB values assuming background luminance of 1, 5, and 10 cd/m^2^ for the Pico Neo 3 based on spectrometer measurements using 4 mm aperture at 0 degrees and (B) 30 degrees. (C) dB values assuming background luminance of 1, 5, and 10 cd/m^2^ for the Oculus Quest 2 based on spectrometer measurements using 4 mm aperture at 0 degrees and (D) 30 degrees. (E) dB values assuming background luminance of 1, 5, and 10 cd/m^2^ for the HTC Vive based on spectrometer measurements using 4 mm aperture at 0 degrees and (F) 30 degrees.

To determine lowest and highest achievable thresholds of the VR devices for perimetric testing scenarios, the empiric luminance measurements in each device were used to calculate minimum and maximum dB with background luminance at 1, 5, and 10 cd/m^2^ at 0 and 30 degrees (Fig 4). Assuming standard background luminance of 10 cd/m^2^ and RGB value of 255 for maximum luminance, HTC Vive showed the best performance with lowest thresholding at 16 dB. With these parameters, Oculus Quest 2 and Pico Neo 3 had minimum thresholds of only 20 dB. In other words, thresholding below 20 dB was not achievable for these commonly used VR devices under these conditions. Each device had higher minimum dB thresholds at 30 degrees in the periphery, with Pico Neo 3 showing the highest minimum threshold of 23 dB.

## Discussion

The current paradigm of diagnosing and monitoring glaucoma faces numerous challenges including available resources and access to visual field testing. The use of VR head-mounted devices presents a number of potential advantages, including improved access to testing, ease of testing for those with mobility issues, and lower cost of testing as compared to conventional bowl perimeters [8]. While the development of VR headsets with the capability for visual field testing has rapidly expanded in recent years, there remains a paucity of evidence on how well VR headsets mimic the critical specifications of conventional bowl perimeters. This review and experimental performance testing sought to evaluate VR-based visual field testing systems in the literature.

Luminance is a photometric measurement of the luminous intensity per unit area of a surface or light source, typically expressed in candelas per square meter (cd/m^2^). Luminance is measured with spectrometers, which calculate luminance based on the received light intensity and angle of observation. Handheld luminance meters offer portable data collection but are generally limited in sensitivity and resolution in light collection and analysis. Spectroradiometers are more commonly used for more precise light measurements in photometric research and evaluating LED performance, as these devices house more sophisticated systems including adjustable apertures, high sensitivity detectors, and advanced analytics [52].

In addition to absolute luminance, contrast is another important parameter in perimetry. While absolute luminance refers to the brightness of the stimuli presented, contrast is the luminance relationship of the stimuli and its background. A maximum of 1:3746 Weber contrast for the conventional automated perimeter was predetermined as there is no theoretical maximum contrast when using luminance increments [53].

Based on our review of VR headsets for perimetry, the majority of VR headsets produced an absolute luminance of <5% of that required to achieve non-inferiority to conventional bowl perimeters. Given that glaucoma patients with severe visual field defects need relatively higher intensities of stimulus for detection, the sub-standard range of luminance that is inherent to VR perimetry would significantly restrict the ability to measure more advanced visual field defects [43]. Therefore, current VR devices can theoretically identify areas of decreased sensitivity, but deeper field defects would be underestimated due to substantial limitations in maximum luminance range compared to that of conventional bowl perimetry [43]. Few non-commercially available devices, such as the VirtualEye system, have reported higher luminance levels approaching the conventional maximum luminance [54]; however, the measurement and reporting of luminance levels are not standardized in these headsets.

In this review, no commercially available VR devices programmed for visual field testing could achieve the full range of Weber contrast of conventional bowl perimeters. One method to try to achieve higher levels of contrast, which deviates from conventional bowl perimetry, is to lower the background luminance. We observed that decreasing this as low as 1 cd/m^2^ still limits the full range of achievable threshold values. While our empirical measurements utilized RGB pixel values and unmodulated luminance output, it is important to note that some VR perimetry platforms may implement software modifications such as bit-stealing, temporal flickering, alpha channel modulation, and pixel subsampling to simulate even lower background luminance. Physiologically, decreasing the background luminance below 3 cd/m^2^ into the mesopic conditions results in activation of both rods and cones, whereas only cones are active in photopic conditions by conventional HFA. In addition, lowering the background luminance may increase the confounding effect of media opacities. VR devices programmed with lower background luminance present a fundamental challenge to accurately compare the results of VR perimetry to conventional perimetry.

Among forty-two studies describing the devices included in our review, only four studies mentioned luminance output as a disadvantage. This highlights the importance of understanding the critical parameters in perimetry, particularly when used to diagnose and monitor disease processes. Additionally, even among commercially available devices, reported luminance varied among studies, likely reflecting differences in measurement methods and variability of individual devices of the same model.

For our experimental measurements, we acquired three VR headsets: Pico Neo 3, Oculus Quest 2, and HTC Vive. Although phone-based headsets were commonly employed, there was a variability of smartphones used, and the viewing experience is generally limited for VR compared to tethered devices.

The empirical measurements using VR headsets established that aperture size significantly affects luminance measurements at all brightness levels. An aperture size of 4 mm was used for the remainder of the study, as the physiologic pupil size is typically 4 mm in standard bowl perimetry, and the Humphrey Field Analyzer is calibrated using a 4 mm-aperture spectrometer.

Measurement of luminance was inconsistently reported in studies to date, with only three named handheld luminance meters in a small proportion of studies. In comparison to high-resolution spectroradiometers, handheld meters significantly overestimate true luminance across all brightness levels. Across all RGB parameters, we showed that there is increasing deviation from true luminance values in more peripheral measurements in a non-linear relationship. Therefore, the application of handheld meters for validating VR devices for perimetry is limited.

For luminance measurements spanning up to 30 degrees peripherally, maximum luminance declined across all brightness levels in VR headsets. This is particularly relevant as conventional bowl perimetry presents stimuli up to 30 degrees to foveal fixation to detect non-central visual field loss. Compared to conventional bowl perimeters that provide curved and uniform illumination field, VR devices contain flat displays viewed through high-powered convex lenses or Fresnel lenses. These lenses introduce optical aberrations, which further impact light transmittance at higher eccentricities and contribute to non-uniform luminance and peripheral dimming. Currently available VR devices or software have not reported any method to compensate for reduced luminance across the testable visual field.

The highest empirical L_max_ measurement was 80 cd/m^2^ with HTC Vive at 0 degrees and RGB value of 255. Under these optimal parameters and with the assumption of standard background luminance of 10 cd/m^2^, the HTC Vive is not able to threshold below 16 dB. Clinically, this perimeter will underestimate severity of visual field loss, as it would cap visual field areas of reduced sensitivity at 16 dB (i.e., it cannot distinguish between loss at 15dB or an absolute loss at <0dB), resulting in an artificially higher or lower mean deviation (MD) value (depending on how the system is programmed for readings between 0 and 15 dB). Additionally, we have shown that other VR devices and peripheral measurements produce even higher, more limited, minimum achievable thresholds.

In summary, we have demonstrated that commercially available VR devices do not meet the standards of conventional bowl perimetry with regards to luminance and thus thresholding capability. There are no devices in the literature that generate sufficient luminance levels to meet non-inferiority. We have also shown that factors such as luminance meter, pupil size, and peripheral stimulus location significantly affect luminance measurements, which have not been previously addressed as potential confounding factors when reporting luminance performance of VR devices. Notably, luminance and stimulus location in degrees from center have a non-linear relationship that require empiric validation and inclusion in calibration.

The challenge may perhaps be related to the adaptation of existing VR devices developed for entertainment for perimetry, re-purposed and re-programmed for a specialized application such as visual field testing. Future development in perimetry using head-mounted devices may benefit from hardware specifications capable of producing the background and full range of stimulus luminance levels, and thus contrast levels, to be able to achieve non-inferior light sensitivity thresholding across the tested field of view.

## Limitations

Luminance data was based on measurement using various photometers and luminance meter devices, many of which are unnamed or not reported in studies or by manufacturers. The different measurements could lead to inconsistent reporting of luminance amongst VR devices. Further study of these VR devices using controlled measurement of luminance would be helpful to understand variance unrelated to the devices. Of note, although stimuli were standardized by RGB values across devices, spectral distribution and color temperature can vary at equal luminance levels, which are confounding factors in luminance perception and stimulus consistency. This variability is not captured by luminance measurements alone, and calibration of these variables across VR devices is recommended. Given that consumer-grade VR devices can employ software and color channel modulation to manipulate perceived luminance, the standardization of VR performance remains an important area of future research. Finally, despite thirty-four devices included in this review, we were able to use luminance data from twenty-four due to unavailable data, limiting the generalizability of our conclusions.

## Conclusion

While VR headsets offer many advantages, we suggest that based on our review, current VR technology does not meet the standard of luminance and contrast sensitivity used in traditional bowl perimetry. Currently available VR technologies will encounter limitations in detecting field loss and progression in moderate to advanced glaucoma cases due to a limited range of maximum luminance, particularly for peripheral stimuli. VR-based perimetry with current displays may have a role in screening for advanced diseases, but rigorous standardization and calibration is required for clinical applications. We suggest further research to evaluate the reliability and accuracy of VR technology in this context.

## Supporting information

S1 File Data_sharing. (ZIP)

S2 File Plots and stats. (R)

S3 File Process handheld photometer data. (R)

S4 File Process spectroradiometer data. (R)

S5 File Coded values vs luminance – HTC Vive. (CSV)

S6 File Coded values vs luminance – Oculus Quest 2. (CSV)

S7 File Coded values vs luminance – Pico Neo 3. (CSV)

S8 File Coded values vs luminance 2 mm aperture. (XLSX)

S9 File Coded values vs luminance 4 mm aperture. (XLSX)

S10 File Coded values vs luminance 8 mm aperture. (XLSX)

S11 File Aperture 4mm. (RDS)

S12 File Screen luminance. (RDS)

S13 File Spectroradiometer. (RDS)
